# Effectiveness and safety of oral HIV preexposure prophylaxis for all populations

**DOI:** 10.1097/QAD.0000000000001145

**Published:** 2016-07-13

**Authors:** Virginia A. Fonner, Sarah L. Dalglish, Caitlin E. Kennedy, Rachel Baggaley, Kevin R. O’Reilly, Florence M. Koechlin, Michelle Rodolph, Ioannis Hodges-Mameletzis, Robert M. Grant

**Affiliations:** a Johns Hopkins Bloomberg School of Public Health, Baltimore, Maryland, USA; b HIV Department, World Health Organization, Geneva, Switzerland; c Medical University of South Carolina, Charleston, South Carolina, USA; d HIV Department, World Health Organization, Switzerland; Gladstone Institutes and the University of California; San Francisco AIDS Foundation, San Francisco, California, USA.

**Keywords:** HIV, HIV prevention, meta-analysis, preexposure prophylaxis, systematic review, tenofovir

## Abstract

**Objective:**

Preexposure prophylaxis (PrEP) offers a promising new approach to HIV prevention. This systematic review and meta-analysis evaluated the evidence for use of oral PrEP containing tenofovir disoproxil fumarate as an additional HIV prevention strategy in populations at substantial risk for HIV based on HIV acquisition, adverse events, drug resistance, sexual behavior, and reproductive health outcomes.

**Design:**

Rigorous systematic review and meta-analysis.

**Methods:**

A comprehensive search strategy reviewed three electronic databases and conference abstracts through April 2015. Pooled effect estimates were calculated using random-effects meta-analysis.

**Results:**

Eighteen studies were included, comprising data from 39 articles and six conference abstracts. Across populations and PrEP regimens, PrEP significantly reduced the risk of HIV acquisition compared with placebo. Trials with PrEP use more than 70% demonstrated the highest PrEP effectiveness (risk ratio = 0.30, 95% confidence interval: 0.21–0.45, *P* < 0.001) compared with placebo. Trials with low PrEP use did not show a significantly protective effect. Adverse events were similar between PrEP and placebo groups. More cases of drug-resistant HIV infection were found among PrEP users who initiated PrEP while acutely HIV-infected, but incidence of acquiring drug-resistant HIV during PrEP use was low. Studies consistently found no association between PrEP use and changes in sexual risk behavior. PrEP was not associated with increased pregnancy-related adverse events or hormonal contraception effectiveness.

**Conclusion:**

PrEP is protective against HIV infection across populations, presents few significant safety risks, and there is no evidence of behavioral risk compensation. The effective and cost-effective use of PrEP will require development of best practices for fostering uptake and adherence among people at substantial HIV risk.

## Introduction

An estimated two million people became infected with HIV in 2014 [1], demonstrating the dire need for more effective, safe, and accessible prevention options. One such promising tool is preexposure prophylaxis (PrEP) – the use of antiretroviral medications by HIV-uninfected individuals to block HIV acquisition. In 2012, the WHO recommended offering oral PrEP containing tenofovir disoproxil fumarate (TDF) among HIV serodiscordant couples and MSM, with the conditionality that demonstration projects were needed to ascertain optimal delivery approaches and target groups [2]. In 2014, these recommendations were integrated into consolidated HIV guidelines for key populations, including a strong recommendation for offering PrEP as a prevention option for MSM [3]. However, as experience with PrEP across populations from clinical trials, demonstration projects, and clinical practice has grown, so has the need to evaluate PrEP among all people at high HIV risk. To date, no systematic assessment of PrEP's effectiveness across populations exists. We conducted this systematic review and meta-analysis of the effectiveness of oral PrEP containing TDF for all people at substantial risk of HIV [4].

## Methods

### Search strategy and inclusion criteria

For inclusion, a study had to: be a randomized controlled trial (RCT), an open-label extension (OLE), or a demonstration project evaluating oral PrEP containing TDF to prevent HIV infection; measure one or more key outcomes, comparing those randomized to PrEP vs. placebo or those receiving PrEP vs. no PrEP use (i.e., delayed PrEP); and be published in a peer-reviewed journal or presented at a scientific conference between 1 January 1990 and 15 April 2015. Key outcomes included: HIV infection, adverse events, antiretroviral drug resistance, reproductive health (hormonal contraception effectiveness and adverse pregnancy-related events), and behavior (condom use and number of sexual partners). We followed Preferred Reporting Items for Systematic Reviews and Meta-Analyses Guidelines for reporting systematic reviews and meta-analyses [5].

Our search strategy included electronic databases, scientific conference websites, and secondary searching of included studies. We searched PubMed, Cumulative Index to Nursing and Allied Health Literature, and Embase using predetermined search terms (available from authors upon request). For conferences, we searched abstracts from the International AIDS Conference (IAC), International AIDS Society (IAS) Conference on HIV Pathogenesis, Treatment, and Prevention, and Conference on Retroviruses and Opportunistic Infections. For IAS/IAC, we searched conferences from 2006 to 2014. For CROI, only abstracts from 2014 to 2015 were publicly available. We also conducted iterative secondary reference searching on all included studies.

### Data abstraction and management

Study authors initially screened titles, abstracts, and study descriptors of identified citations. Two independent reviewers screened the remaining citations, obtained full text articles, and independently extracted data from included studies using standardized forms. Differences in data extraction were resolved through consensus. For RCTs, we evaluated risk of bias using the Cochrane Collaboration's risk assessment tool [6].

### Analysis

We conducted meta-analysis using random-effects models with Comprehensive Meta-Analysis v3.0, checking sensitivity by running primary analyses with and without certain studies with predetermined characteristics, including adherence. In meta-analysis, we stratified by study design (e.g. RCT or observational) and comparator (e.g., placebo or delayed PrEP).

Because this review covered multiple populations, drug regimens, dosing schemes, and comparators, we conducted subgroup analyses identified a priori, including biological sex, age (<25 or ≥25 years), primary mode of sexual HIV acquisition (rectal or penile/vaginal exposure), adherence level, PrEP dosing (daily or intermittent), and regimen [TDF alone or in combination with emtricitabine (FTC/TDF)]. We performed subgroup analyses only among studies presenting stratified data; participant-level data were not analyzed. We defined studies’ overall adherence level based on the percentage of HIV-negative participants receiving PrEP with discernible levels of study medication in their blood when sampled. Studies presenting this information, usually as part of a case-control or case-cohort analysis, also presented results of detectable drug levels found among seroconverters (Table 9S). If studies did not report blood-based drug detection, they were excluded from this analysis. Trial-level adherence levels were divided into three categories with ‘high’ adherence defined as more than 70%, ‘moderate’ as 41–70%, and ‘low’ as 40% or less drug detection. When possible we used Comprehensive Meta-Analysis v3.0 to conduct bivariate method of moments random-effects meta-regression to evaluate whether variables moderated the effect of PrEP on reducing risk of HIV infection.

## Results

### Description of included studies

**Fig. 1:**
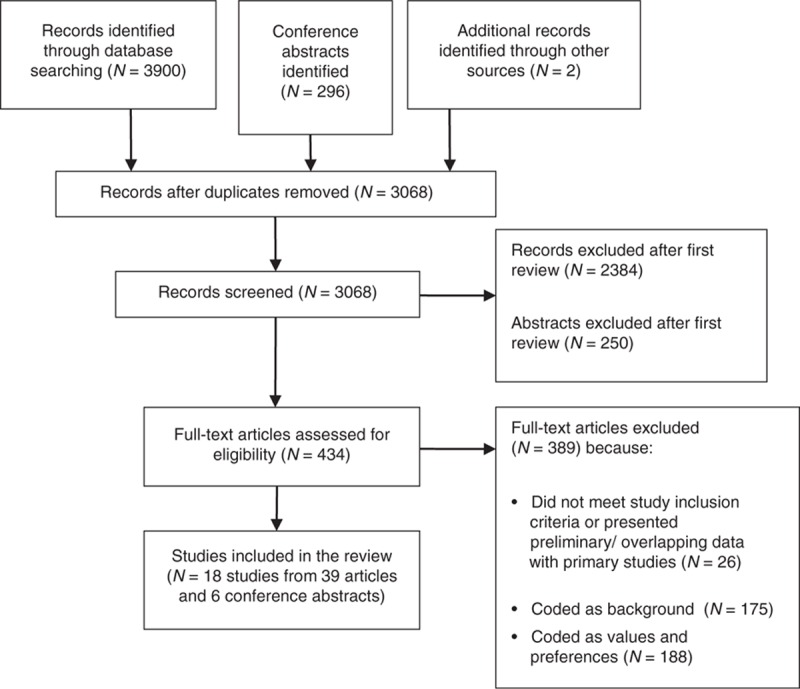
Disposition of study citations during searching and screening process.

Of 3068 citations screened, 39 articles and six conference abstracts covering 18 PrEP-related studies were included (Fig. 1). We included 15 RCTs and three observational OLE or demonstration projects (Table 1). Seven RCTs were double-blind placebo-controlled trials evaluating the efficacy and safety of daily oral PrEP [8,18,24,32,45,47,49]. Two studies randomized participants to receive immediate or delayed PrEP [13,43], and one study compared daily PrEP with both placebo and ‘no-pill’ arms [42]. Several trials examined alternative PrEP dosing strategies [21,30,31], including nondaily PrEP (taken before and after sexual intercourse). Two open-label RCTs compared different PrEP regimens and dosing strategies with no placebo arm [7,40]. Three demonstration projects and OLE continuations from previous RCTs were also included [11,29,41].

Included studies involved 19 491 participants, of whom 11 901 received active PrEP, with follow-up times ranging from 24 weeks to 5 years. Populations included people who inject drugs, serodiscordant couples, MSM and transgender women, women, and heterosexual men. Trials occurred in low, middle and high-income settings. Overall RCTs were judged to have low risk of bias (Table 1S). Several studies had unclear risk for reporting bias, either because study protocols were not publicly accessible or available data included only preliminary results. Overall adherence levels, as measured by drug detection, were exceptionally low in two studies, FEM-PrEP and VOICE [18,47], which compromised their ability to accurately assess PrEP effectiveness.

### HIV infection

**Fig. 2:**
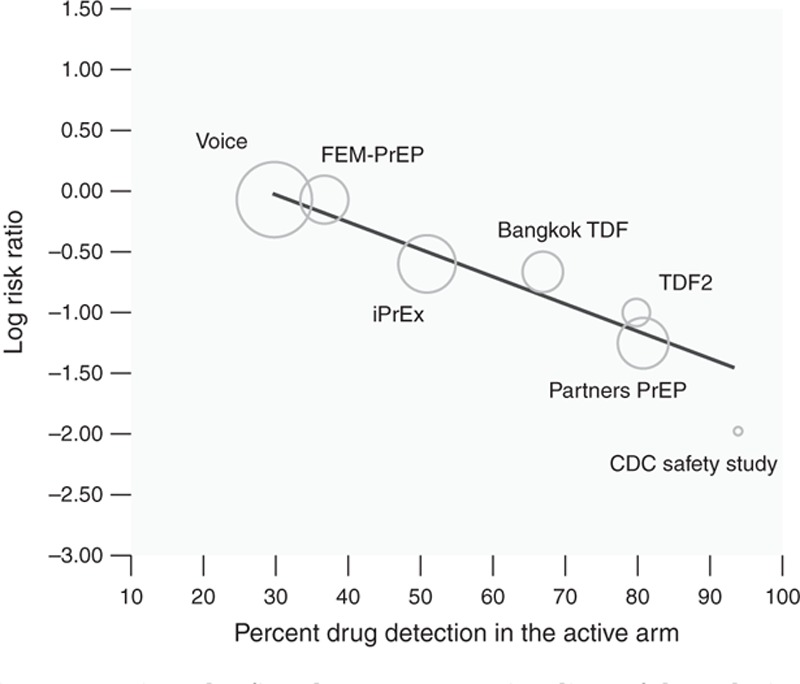
Depicts the fitted meta-regression line of the relationship between trial-level PrEP adherence and PrEP's effectiveness in preventing HIV acquisition.

HIV infection was measured in 11 RCTs comparing PrEP with placebo, three RCTs comparing PrEP with no PrEP (e.g. delayed PrEP or ‘no pill’), and three observational studies. Across placebo-controlled trials (Table 2a, Fig. 2), results from meta-analysis demonstrated a 51% reduction in risk of HIV infection comparing PrEP with placebo [risk ratio = 0.49, 95% confidence interval (CI): 0.33–0.73, *P* = 0.001]. Results from meta-regression suggest adherence was a significant moderator of PrEP effectiveness (regression coefficient = −0.02, *P* < 0.001) (Table 2a, Fig. 2). When stratified by adherence, overall heterogeneity was greatly reduced. PrEP was most effective in studies with high adherence, where HIV infection risk was reduced by 70% (risk ratio = 0.30, 95% CI: 0.21–0.45, *P* < 0.001). PrEP also significantly reduced infection risk in studies with moderate adherence levels, but showed no effect in studies with low adherence (risk ratio = 0.95, 95% CI: 0.34–1.23, *P* = 0.70). In studies comparing immediate with delayed PrEP [42,43], PrEP was protective against HIV infection (risk ratio = 0.15, 95% CI: 0.05–0.46, *P* = 0.001). Reductions in HIV incidence were also seen in observational studies (Table 2b) [11,29,41].

Trial-level adherence is measured along the x-axis as the percentage of participants who received active study drug and had detectable levels of either TDF or FTC in their blood during the study. Effectiveness in preventing HIV acquisition is measured along the y-axis as a log-risk ratio. Each circle represents a study, and the size of the circle is directly proportional to the study's size and weight in meta-regression. PrEP, preexposure prophylaxis; TDF, tenofovir disoproxil fumarate.

When stratified by mode of acquisition, PrEP showed similar effectiveness across groups (coefficient = 0.47, *P* = 0.36) (Table 2a). The relative risk for HIV infection comparing PrEP with placebo for rectal exposure was 0.34 (95% CI: 0.15–0.80, *P* = 0.01) and 0.54 (95% CI: 0.32–0.90, *P* = 0.02) for penile/vaginal exposure. Across other stratifications, PrEP remained significantly protective against HIV infection. No significant differences in PrEP effectiveness were seen between sexes, regimens, and dosing, although effectiveness data for intermittent PrEP were limited to one study. For PrEP regimens, results from meta-regression suggest TDF PrEP was as effective as FTC/TDF PrEP (meta-regression *P* value = 0.88); this finding remained consistent when stratified by sex. Similarly, the Partners PrEP Study Continuation found no difference in HIV-prevention effectiveness comparing daily FTC/TDF with TDF [40].

Three studies provided age-stratified data (<25 years and ≥25 years) [18,24,32]. In meta-regression, age did not moderate the relationship between PrEP and HIV infection (coefficient = 0.45, *P* = 0.29, Table 2a); however, in stratified analysis PrEP was not statistically effective for younger participants (risk ratio = 0.71, 95% CI: 0.47–1.06, *P* = 0.07). Several studies noted that younger participants had poorer adherence compared with older participants [8,29]. Therefore, while age may not moderate the relationship between PrEP and HIV infection, low adherence could explain diminished effectiveness among young populations.

We also evaluated age and sex-stratified data, which were reported in two studies, to evaluate PrEP effectiveness among young women. PrEP was not effective in preventing HIV infection among women aged less than 25 years in FEM-PrEP [12] but did effectively reduce infection among women aged less than 30 years in Partners PrEP [38].

### Adverse events

Ten placebo-controlled RCTs presented data on any adverse event. Across studies, proportions of adverse events comparing PrEP with placebo were similar (odds ratio = 1.01, 95% CI: 0.99–1.03, *P* = 0.27). No differences were seen across subgroups based on mode of acquisition, adherence, sex, drug regimen, dosing, or age (Table 3). Comparing immediate with delayed PrEP, two studies reported occasional PrEP interruptions because of medical events, such as gastrointestinal symptoms, but noted PrEP was reinitiated in most participants without event recurrence [29,43]. Regarding drug regimen, the Partners PrEP Continuation Study found no significant difference in adverse events comparing FTC/TDF and TDF [20].

Eleven placebo-controlled RCTs presented results on any grade 3 or 4 adverse event, proportions of which did not differ between PrEP and placebo groups (risk ratio = 1.02, 95% CI: 0.92–1.13, *P* = 0.76). No statistically significant differences were seen across subgroups (Table 3). Several studies reported small, subclinical decreases in renal function among PrEP users [9,28]. Although function mostly returned to normal following PrEP discontinuation. Additionally, some studies reported small, subclinical decreases in liver function [8,18], and bone mineral density [15,44] while taking PrEP.

### Drug resistance

Six trials measured and reported cases of TDF or FTC drug resistance, identified using standardized clinical genotypic laboratory assays [8,18,24,32,45,47]. Results from ultrasensitive analyses were excluded because of lack of validation for clinical use. Within these trials, eight (18%) HIV infections with mutations conferring resistance to TDF or FTC occurred among 44 individuals acutely HIV-infected at enrollment, comprising two resistant infections among those randomized to placebo and six among those randomized to PrEP. In addition, six (2%) TDF or FTC drug-resistant infections occurred out of 533 cases of incident HIV infection postrandomization across study arms (Table 2S), including five FTC mutations among those randomized to PrEP and one mutation among those randomized to placebo.

Additional HIV infections had resistance to drugs unrelated to PrEP, likely because of primary drug resistance. Definitively distinguishing between primary and secondary (PrEP-selected) drug resistance was not possible for most infections.

When comparing PrEP (any regimen) with placebo, risk of developing FTC and/or TDF mutations was significantly higher in PrEP vs. placebo groups (risk ratio = 3.34, 95% CI: 1.11–10.06, *P* = 0.03, Table 3S) among those acutely infected at enrollment. When stratified by PrEP regimen, the risk of having an FTC-related mutation for those acutely infected at enrollment was significantly higher among participants randomized to receive FTC/TDF as compared with placebo (risk ratio = 3.72, 95% CI: 1.23–11.23, *P* = 0.02, Table 3S). Risk of having a TDF-related mutation was not statistically different between PrEP and placebo, regardless of PrEP regimen, among those acutely infected at enrollment.

Among participants who seroconverted postrandomization, FTC, or TDF resistant infections were uncommon, leaving little power to assess relative risk. With TDF PrEP, no seroconverters had resistance to tenofovir in either placebo or active arms. Across PrEP regimens, statistically insignificant increases in the proportion of new infections with FTC or TDF-related mutations comparing PrEP with placebo (risk ratio = 3.14, 95% CI: 0.53–18.52, *P* = 0.21, Table 3S) were found among those who seroconverted postrandomization. Results remained insignificant when stratified by mutation type and PrEP regimen.

### Reproductive health

FEM-PrEP and Partners PrEP reported hormonal contraception effectiveness comparing participants receiving PrEP vs. placebo [20,39]. In FEM-PrEP, hormonal contraception use was required for trial participation. In Partners PrEP, hormonal contraception use was not required, but monthly study visits included contraceptive counseling and free on-site contraception access.

When comparing pregnancy rates among contraceptive users receiving PrEP and placebo, results from raw data demonstrated higher pregnancy rates for those receiving PrEP (Table 4S). However, because of confounding across study arms we present separate adjusted pregnancy rates comparing PrEP and placebo groups (Table 5S). In both FEM-PrEP and Partners PrEP, treatment assignment became an insignificant predictor of pregnancy when adjusted for confounders [20,39]. Owing to differing analytic comparisons, synthesis of adjusted data was infeasible. Both studies noted higher pregnancy incidence among women taking combined oral contraceptives compared with injectable or implantable methods.

FEM-PrEP and Partners PrEP also evaluated effects of PREP on adverse pregnancy-related events (Table 6S). Study drug was discontinued for women once pregnancy was confirmed across trials; therefore, the effect of PrEP throughout pregnancy duration was not assessed. Across studies risk of adverse pregnancy-related events did not differ between PrEP and placebo arms (risk ratio = 1.25, 95% CI: 0.64–2.45, *P* = 0.52), and results remained insignificant when stratified by adherence and PrEP regimen. In the Partners PrEP Study Continuation, pregnancy loss frequency was similar between PrEP regimens [35].

### Sexual behavior

Condom use was reported in five RCTs comparing PrEP with placebo [18,24,32,45,48], three RCTs comparing PrEP with no-PrEP [12,42,43], one observational study [29], and one longitudinal analysis comparing outcomes from the placebo-controlled phase and OLE continuation [36]. Owing to differences in condom use measurement across studies, meta-analysis was infeasible. However, studies consistently showed no difference in condom use across arms (Table 7S), and some even showed increases in condom use throughout trial duration. Among studies comparing PrEP with no-PrEP, which more accurately reflect real-life scenarios than placebo-controlled RCTs, studies similarly found either no change in condom use across arms or slight increases in condom use over time [12,13,42]. Notably in PROUD, investigators used incident sexually transmitted infections (STIs) as a biological proxy for noncondom sexual intercourse and found similar rates across immediate and delayed PrEP arms [13]. The longitudinal Partners PrEP analysis comparing placebo-controlled RCT with OLE continuation periods found trends toward decreasing frequency of noncondom intercourse with HIV-positive study partners but also noted increased frequency of noncondom intercourse with outside partners over time [36].

Eight placebo-controlled trials, two RCTs comparing PrEP with no-PrEP, and three observational studies examined number of sexual partners. Like condom use, differing measurements precluded meta-analysis; however, results across studies found no evidence that PrEP impacted participants’ reported number of sexual partners (Table 8S). Among placebo-controlled RCTs, many found small reductions in sexual partners reported over time [10,18,45] or no change across study arms [24,31,32]. The IAVI Kenya study was the only trial to find an increase in sexual partners from baseline to follow-up, although investigators noted the possibility of partner underreporting at baseline [30]. When comparing PrEP with no-PrEP, studies either found decreases in reported number of sexual partners [12] or no change from baseline to follow-up among participants [13]. Observational studies showed similar null results [11,29].

## Discussion

### Evidence summary and implications

This review evaluated the effect of oral PrEP containing TDF in 15 RCTs and three observational studies. PrEP was effective in reducing risk of HIV acquisition across types of sexual exposure, sexes, PrEP regimens, and dosing schemes. Studies have suggested a possible biological mechanism for different rates of protection according to primary transmission route, in that higher rates of drug concentration have been found in rectal tissue compared with vaginal; [50,51]; however, we found no such differences in protective effects. In our analyses, trial-level adherence moderated the impact of PrEP on HIV acquisition, as PrEP was more effective in reducing risk of HIV infection with higher levels of PrEP adherence. Overall, the level of effectiveness within each study was similar to the proportion of people in the active arm who had PrEP drug detected, indicating that PrEP is highly efficacious when used.

The finding that TDF and FTC/TDF have comparable effectiveness in meta-analysis is consistent with two clinical placebo-controlled trials that compared the regimens directly in heterosexual populations [32,47], and with one study comparing single and dual-agent PrEP [40]. TDF PrEP for heterosexual populations may be attractive because of its comparable effectiveness, lower cost, greater availability, and lower risk of drug resistance [34]. Only one safety study evaluated TDF PrEP among MSM; other trials among MSM used FTC/TDF PrEP.

For young women, one study found PrEP was effective in reducing HIV infection and another study found no effect, most likely associated with differing levels of adherence. Results from one open label study demonstrate that young women can maintain high levels of PrEP use when aware PrEP is effective [7]. A more recent OLE completed after our search period also found that women can be highly adherent to PrEP [52]. Despite this evidence, gaps exist in knowing how PrEP will be perceived and used among young people in real-world settings, and research is needed to understand what supportive interventions, tailored to young people's needs, could be implemented in combination with PrEP [53]. Promising approaches include providing information about how well PrEP works when used properly; building community support for PrEP; allowing choice in contraceptive use; and combining PrEP programs with social marketing campaigns and adherence support programs [54].

Regarding safety, PrEP showed no evidence of increased proportion of adverse events. However, two studies reported small decreases in renal function among those taking PrEP [9,28]. PrEP programs have used relatively intensive monitoring of renal function, including frequent creatinine testing, which may or may not be required to assure safety. Several trials demonstrated a small decrease in bone mineral density during the first 24 weeks of PrEP use that did not progress thereafter, including one study published after our search that showed small, reversible decreases in bone mineral density among African women [55]. Given that HIV infections occurring in the absence of PrEP would require lifelong antiretroviral therapy, which is associated with a three to four-fold greater loss of bone mineral density compared with PrEP [56], and HIV has direct toxicity to bone [57], this presents a favorable risk benefit ratio.

The risk of tenofovir or FTC resistance during use of PrEP was low. In meta-analysis, participants randomized to PrEP had a higher risk of resistance compared with placebo among those acutely HIV-infected when starting PrEP, with more cases of resistance occurring to FTC than TDF. This is consistent with results from the Partner's PrEP Study Continuation that compared the regimens directly [34]. The risk of drug resistance with PrEP has to be weighed with overall benefits [58]. If PrEP had been withheld, more HIV infections would have occurred, which would require life-long therapy with an annual risk of drug resistance varying between 5 and 20%. As such, levels of drug resistance occurring by preventing HIV infection with PrEP are expected to be less than if HIV is left unchecked, as predicted by mathematical modeling [59,60]. How implementing PrEP on a large scale affects resistance overall is unknown, and active surveillance is warranted.

Regarding sexual behaviors, we found no evidence that PrEP led to risk compensation; however, recent results from real-world PrEP implementation in San Francisco found a relatively high incidence of STIs and a 41% decrease in reported condom use among a subset of PrEP users [61]. RCTs are not well suited to assess risk compensation as participants’ perceptions of protection are unknown, particularly as participants are unaware whether they are receiving an effective, active agent [62]. The lack of risk compensation seen in the OLE studies provides better evidence regarding risk compensation, as these scenarios more closely mirror real-world use. However, these participants also received intensive behavioral counseling and previously served as trial participants, suggesting their behavior might be dissimilar to those taking PrEP outside of a research setting. The continued reduction in sexual risk behaviors seen across the OLE studies and demonstration projects speaks to the potential effectiveness of providing counseling and other prevention options within the context of PrEP implementation.

Regarding pregnancy, PrEP does not appear to affect hormonal contraception effectiveness, although two studies found trends toward higher rates of pregnancy among oral contraceptive users who took PrEP. Oral PrEP was not associated with increased adverse pregnancy-related events among women taking PrEP during early pregnancy.

### Limitations

This review has several limitations. Despite comprehensive searching, our strategy may have failed to identify eligible studies. For included studies, we made efforts to contact study authors for clarifications when necessary, but not all investigators were reachable. Behavioral outcomes were mostly based on self-report, although two studies [24,32] also reported decreasing rates of STIs and one study reported decreases in acute HIV infection prevalence commensurate with reported safer behavior [27]. Additionally, several outcomes (drug resistance and pregnancy outcomes) had few numbers of absolute events, thus leading to imprecision of combined effect sizes. Although we assessed PrEP's effectiveness in preventing sexual acquisition of HIV, we did not examine parenteral transmission of HIV as only one study, the Bangkok Tenofovir Study, involved people who inject drugs. Finally, this review synthesized results from trial-level data only. Although the statistical techniques we employed allowed us to draw inferences about factors affecting PrEP effectiveness overall, not analyzing individual data prevented us from drawing definitive conclusions about individual circumstances of PrEP use and effectiveness.

In conclusion, findings demonstrate oral PrEP containing TDF is effective in reducing risk of HIV infection among various populations. There is little evidence of risk compensation and adverse safety events. For outcomes with few events, including drug resistance and reproductive health outcomes, active surveillance is needed. Surveillance for safety is also warranted for PrEP users not adequately represented in clinical trials, including adolescents, people with underlying comorbidities affecting renal function, and transgender people. PrEP uptake and adherence among people at substantial risk for HIV are key determinants of impact. Based on a collection of substantial evidence, including results from this analysis, a review of PrEP acceptability [63], and cost/feasibility considerations, WHO expanded its 2014 PrEP recommendation to support offer of PrEP to all populations as substantial HIV risk [4]. Best practices for optimizing PrEP delivery based on clinical practice and evidence are now needed.

## Acknowledgements

We thank Peter Godfrey-Faussett, Tim Farley, and Rosalind Coleman for their feedback and insights. We also thank Salim Abdool Karim, Waffa El-Sadr, and the entire WHO Technical Working Group on PrEP for their guidance. We also thank all PrEP trial investigators who provided additional data and clarification during the review process.

WHO/Bill & Melinda Gates Foundation provided funding for this project.

R.B. and K.O. conceived and commissioned the review, including developing the research question, outcomes of interest, and inclusion criteria. V.F. and S.D. conducted the literature search, screening, and data abstraction. F.K. also conducted citation screening. M.R. and F.K. provided feedback on protocol development and helped organize review logistics. I.H.M. abstracted data relating to bone fracture rates. C.K. contributed to the protocol design and conducted several previous reviews on PrEP effectiveness in subpopulations with V.F., including data abstraction that was used in the current review. V.F., S.D., and R.G. analyzed data. C.K. also provided meta-analysis guidance. V.F. wrote the first draft of this manuscript. R.G. aided in calculation and interpretation of drug resistance outcomes and provided overall guidance for the review and manuscript preparation. All authors provided feedback on drafts of the manuscript and approved of the final version.

### Conflicts of interest

There are no conflicts of interest.

## Supplementary Material

Supplemental Digital Content

## Figures and Tables

**Table 1:** List of included studies. Tables for ‘effectiveness and safety of oral HIV preexposure prophylaxis for all populations: a systematic review and meta-analysis’.

Study	Study design	PrEP regimen	PrEP dosing and comparison	Trial-level adherence	Primary mode of HIV acquisition	Location	Study population	Biological sex and age distribution	Number of participants
ADAPT HPTN 067 [7]	CT	FTC/TDF	Daily, time and event-driven PrEP	93.4% to 53.1% (varied by week and study group)	Vaginal	South Africa	Women	Median age: 26 years (range 18–52)	179
								Sex: 100% women	
Bangkok Tenofovir Study 8,9,10	RCT	TDF	Daily PrEP to placebo	67%	Vaginal/penile^a^	Thailand	People who inject drugs	Median age: 31 years (range: 20–59)	2413
								Sex: 80% men	
Bangkok Tenofovir OLE [11]	Cohort	TDF	Daily TDF	Not reported	Vaginal/penile	Thailand	People who inject drugs	Median age: 39 years	787
								Sex: 80% men	
CDC Safety Study 12,13,14,15	RCT	TDF	Immediate/delayed PrEP to immediate/delayed placebo	94%	Rectal	United States	MSM	Age range: 18–60 years	400
								Sex: 100% men	
FEM-PrEP 16,17,18,19,20	RCT	FTC/TDF	Daily PrEP to placebo	37%	Vaginal	Tanzania, South Africa, and Kenya	Women	Median age: 24.2 years (range: 18–35)^b^	2056
								Sex: 100% women	
Ipergay [21]	RCT	FTC/TDF	Intermittent PrEP to placebo	Not reported	Rectal	France and Canada	MSM	Age not reported	400
								Sex: 100% men	
iPrEx 22,23,24,25,26,27,28	RCT	FTC/TDF	Daily PrEP to placebo	51%	Rectal	Peru, Ecuador, South Africa, Brazil, Thailand, and United States	MSM and transgender women	Age range: 18–67 years^b^	2499
								Sex: 100% male at birth; 1% female gender identity	
iPrEx/US-based studies OLE [29]	Cohort	FTC/TDF	Daily PrEP to no PrEP use	71%	Rectal	Peru, Ecuador, South Africa, Brazil, Thailand, and United States	MSM and transgender women	Age: 18–24 years (20%)	1603
								25–29 years (27%)	
								30–39 years (31%)	
								≥40 years (22%)	
								Sex: 100% men	
IAVI Kenya Study [30]	RCT	FTC/TDF	Daily/intermittent PrEP to daily /intermittent placebo	Not reported	Rectal	Kenya	MSM and FSW	Mean age: 26 years (range: 18–49)	72
								Sex: 67 men; 5 women	
IAVI Uganda Study [31]	RCT	FTC/TDF	Daily/intermittent PrEP to daily/intermittent placebo	Not reported	Vaginal/penile	Uganda	Sero-discordant couples	Mean age: 33 years (range: 20–48)	72
								Sex: 50% women; 50% men	
Partners PrEP Study [32–39]	RCT	FTC/TDF and TDF (two active arms)	Daily PrEP to placebo	81%	Vaginal/penile	Kenya and Uganda	Sero-discordant couples	Age range: 18–45 years^b^	4747 couples
								Sex: 61–64% men (depending on group assignment)	
Partners PrEP Study Continuation [40]	RCT	FTC/TDF and TDF (two active arms)	Daily TDF to FTC/TFC	89% (month 1) to 65% (month 36)	Vaginal/penile	Kenya and Uganda	Sero-discordant couples	Age range: 28–40 years	4410 couples
								Sex: 62–64% men (depending on group assignment)	
Partners Demonstration Project [41]	Cohort	FTC/TDF	Daily PrEP	Not reported	Vaginal/penile	Kenya and Uganda	Sero-discordant couples	Age and sex not reported	1013 couples
Project PrEPare [42]	RCT	FTC/TDF	Daily PrEP to placebo and to ‘no pill’	63.2% (week 4) to 20% (week 24)	Rectal	United States	Young MSM	Median age: 19.97 years (range: 18–22)	58
								Sex: 100% men	
PROUD [43]	RCT	FTC/TDF	Immediate PrEP to delayed PrEP	Not reported	Rectal	England	MSM	Median age: 35 years	545
								Sex: 100% men	
TDF2 44,45,46	RCT	FTC/TDF	Daily PrEP to placebo	80%	Vaginal/penile	Botswana	Heterosexual men and women	Age range: 18–39 years	1219
								Sex: 54.2% men; 45.8% women	
VOICE [47]	RCT	FTC/TDF and TDF (two active arms)	Daily PrEP to placebo	30%	Vaginal	South Africa, Uganda, and Zimbabwe	Women	Median age: 24 years (range: 18–40)	4969
								Sex: 100% women	
West African Safety Study [48,49]	RCT	TDF	Daily PrEP to placebo	Not reported	Vaginal	Nigeria, Cameroon, and Ghana	Women	Age range: 18–34 years	936
								Sex: 100% women	

^a^Five percentage of male participants in the Bangkok Tenofovir Study reported sexual intercourse with a male partner in the past 12 weeks at baseline.

^b^The Partners PrEP Study, iPrEx, and FEM-PrEP included data for participants aged <25 years and ≥25 years. The age stratified data included in these studies comprises the sub-group analysis presented in Table 2a. CDC, Centers for Disease Control and Prevention; FTC, emtricitabine; HPTN, HIV Prevention Trials Network; IAVI, International AIDS Vaccine Initiative; OLE, open-label extension; PrEP, preexposure prophylaxis; RCT, randomized controlled trial; TDF, tenofovir disoproxil fumarate.

**Table 2:** Meta-analysis results assessing the effectiveness of preexposure prophylaxis in preventing HIV acquisition across subgroups and metaregression results assessing the impact of subgroup characteristics on effectiveness.

		Results from meta-analysis				Results from metaregression		
Analysis	No. of studies	Total *N*	Risk Ratio (95% CI)	*P* value	*I*^2^	Meta-regression (MR) coefficient	MR standard error	MR *P* value
RCTs comparing PrEP with placebo								
Overall^a^	10	17 423	0.49 (0.33–0.73)	0.001	70.9			
Mode of Acquisition								
Rectal	4	3166	0.34 (0.15–0.80)	0.01	29.1	*ref*		
Vaginal/penile^b^	6	14 252	0.54 (0.32–0.90)	0.02	80.1	0.47	0.51	0.36
Adherence								
High (>70%)	3	6149	0.30 (0.21–0.45)	<0.001	0.0	−1.14	0.23	<0.001
Moderate (41–70%)	2	4912	0.55 (0.39–0.76)	<0.001	0.0	−0.55	0.21	0.01
Low (≤40%)	2	5033	0.95 (0.74–1.23)	0.70	0.0	*ref*		
Biological sex^c^								
Men	7	8704	0.38 (0.25–0.60)	<0.001	34.5	*ref*		
Women	6	8714	0.57 (0.34–0.94)	0.03	68.3	0.46	0.35	0.19
Age								
<25 years	3	2997	0.71 (0.47–1.06)	0.09	20.5	*ref*		
≥25 years	3	6291	0.45 (0.22–0.91)	0.03	72.4	0.45	0.42	0.29
Drug regimen^d^								
TDF	5	8619	0.49 (0.28–0.86)	0.001	63.9	*ref*		
FTC/TDF	7	11 381	0.51 (0.31–0.83)	0.007	77.2	0.06	0.40	0.88
Drug dosing^e^								
Daily	8	16 951	0.54 (0.36–0.81)	0.003	73.6	*ref*		
Intermittent	1	400	0.14 (0.03–0.63)	0.01	0.0	−1.32	0.90	0.14
RCTs comparing PrEP to no PrEP								
Overall	2	723	0.15 (0.05–0.46)	0.001	0.0			

Table 2b HIV infection outcomes for observational studies.				
Study	Study N	HIV Incidence rate no PrEP	HIV Incidence rate OLE PrEP users	Comparison
Bangkok tenofovir OLE	787	0.7 infections per 100 PY (95% CI: 0.5–1.0)	0.5 infections per 100 PY (95% CI: 0.02–2.3)	Placebo arm of trial to OLE
iPrEx OLE	1603	2.6 infections per 100 PY (95% CI 1.5–4.5)	1.8 infections per 100 PY (95% CI 1.3–2.6)	Non-PrEP users in OLE to PrEP users in OLE
Partners demonstration	1013	5.3 infections per 100 PY (95% CI 3.2–7.6)	0.2 infections per 100 PY (95% CI 0.0–1.3)	Simulated counterfactual to OLE

^a^Modified intent-to-treat (MITT) analyses are presented.

^b^The Bangkok Tenofovir Study contributed data to the penile/vaginal sexual exposure analysis as most participants reported engaging in heterosexual sex (although infections could have also been because of parenteral transmission).

^c^Study populations comprising men and women were disaggregated by sex for this analysis.

^d^Studies comparing more than one PrEP regimen contributed to both TDF and FTC/TDF groups (data were disaggregated by regimen).

^e^The IAVI Kenya study was omitted from this analysis because the trial assessed both daily and intermittent PrEP but it is unclear in which placebo arm (daily or intermittent) the one HIV infection occurred. FTC, emtricitabine; OLE, open-label extension; PrEP, preexposure prophylaxis; PY, person year; TDF, tenofovir disoproxil fumarate.

**Table 3:** Meta-analysis results for effects of preexposure prophylaxis on any adverse event.

	Any adverse event				Any grade 3 or 4 adverse event			
Analysis	No. of studies	Pooled risk ratio (95% CI)	*P* value	*I*^2^	No. of studies	Pooled risk ratio (95% CI)	*P* value	*I*^2^
RCTs comparing PrEP with placebo								
Overall	10	1.01 (0.99–1.03)	0.27	38.1	11^a^	1.02 (0.92–1.13)	0.76	16.5
Mode of acquisition								
Rectal	3	1.01 (0.97–1.06)	0.60	6.0	5	1.09 (0.84–1.41)	0.52	19.0
Vaginal/penile	7	1.01 (0.99–1.04)	0.39	51.6	6	1.00 (0.88–1.15)	0.96	28.9
Adherence								
Low	2	0.97 (0.87–1.08)	0.60	85.6	2	1.08 (0.71–1.64)	0.71	58.0
Medium	2	1.01 (0.98–1.04)	0.46	13.9	2	0.95 (0.82–1.10)	0.48	0.0
High	2	1.02 (0.99–1.04)	0.23	28.4	3	1.05 (0.78–1.39)	0.76	51.9
Biological sex								
Men	2	1.00 (0.98–1.03)	0.85	0.0	4	1.07 (0.83–1.39)	0.59	22.8
Women	3	1.00 (0.92–1.07)	0.92	80.2	2	1.08 (0.71–1.64)	0.71	58.0
Drug regimen								
TDF	4	0.98 (0.92–1.04)	0.47	88.5	3	0.95 (0.80–1.13)	0.56	54.1
FTC/TDF	8	1.02 (1.00–1.04)	0.06	0.0	10	1.07 (0.94–1.21)	0.32	17.4
Drug dosing								
Daily	9	1.00 (0.97–1.03)	0.78	65.6	9	1.01 (0.91–1.13)	0.81	21.2
Intermittent	3	1.05 (0.99–1.11)	0.14	0.0	3	1.14 (0.60–2.18)	0.70	0.0
Age	No safety data stratified by age				No safety data stratified by age			
RCTs comparing PrEP to no PrEP								
Overall	Data not reported for PROUD and CDC Safety Study				Data not reported for PROUD; CDC Study included in PrEP vs. placebo analysis			

^a^The FEM-PrEP study did not present results for the outcome ‘any grade 3 or 4 event.’ For this analysis, results from the outcome ‘any serious adverse event’ were used. PrEP, preexposure prophylaxis.
