# Supplementary material for: Effectiveness and safety of oral HIV preexposure prophylaxis for all populations
Source: AIDS. 2016 Jul 13;30(12):1973–83. doi: 10.1097/QAD.0000000000001145 (PMC4949005; doi:10.1097/QAD.0000000000001145)
Supplement: Supplemental Digital Content [file aids-30-1973-s001.docx]

**Effectiveness and safety of oral HIV pre-exposure prophylaxis (PrEP) for all populations: A systematic review and meta-analysis**

**Supplementary Appendix**

Contents

[Study Protocol 2](#_Toc445111845)

[Table 1S. Quality assessment of included studies (risk of bias) 6](#_Toc445111846)

[Figure 1S. Forest plot for overall analysis of PrEP and HIV Infection 7](#_Toc445111847)

[Table 2S. Drug resistant mutations identified in each study among seroconverters 8](#_Toc445111848)

[Table 3S. Meta-analysis results for PrEP and drug resistance among seroconverters 9](#_Toc445111849)

[Table 4S. Summary of pregnancies rates in studies reporting contraceptive effectiveness 10](#_Toc445111850)

[Table 5S. Summary of results for PrEP and hormonal contraception effectiveness 10](#_Toc445111851)

[Table 6S: Meta-analysis results for effect of PrEP on pregnancy-related adverse events 11](#_Toc445111852)

[Table 7S: Summary of changes on condom use across PrEP studies 12](#_Toc445111853)

[Table 8S: Summary of changes in number of sexual partners across PrEP studies 13](#_Toc445111854)

[Table 9S. Drug detection among seroconverters and HIV-negative controls randomized to active study drug 14](#_Toc445111855)

# Study Protocol

**Oral pre-exposure prophylaxis (PrEP) for people at substantial risk of HIV infection:**

**A systematic review and meta-analysis**

**Background**

Pre-exposure prophylaxis (PrEP) is the use of a retroviral drug by HIV-uninfected individuals to block the acquisition of HIV infection. Over the past 5 years, several randomized trials have demonstrated varying levels of effectiveness of oral PrEP in a variety of populations worldwide. In 2012, WHO developed guidance which recommended PrEP for use amongst serodiscordant couples, men who have sex with men, and transgender people with the conditionality that demonstration projects were needed to ascertain optimal delivery approaches and target groups^1^. In 2014, WHO developed consolidated HIV guidelines for key populations, including men who have sex with men, people who inject drugs, sex workers, and prisoners^2^. However, no recommendation on the use of PrEP was made for female sex workers and people who inject drugs, given a lack of evidence and ongoing community consultations. Additionally, no recommendations have been made for other populations who could benefit from oral PrEP, including women and heterosexual men at elevated risk of HIV acquisition. As trial results of PrEP across different populations have become available, WHO is ready to consider the evidence and develop guidance for PrEP among all populations at substantial risk of HIV infection.

The goal of this work is to conduct a systematic review and meta-analysis of the effectiveness of PrEP for people at substantial risk of HIV infection, and to conduct a systematic literature review of community values and preferences for PrEP.

**PICO question**

**PICO 1:** Should oral PrEP (containing tenofovir (TDF)) be offered as an additional prevention choice for people at substantial risk of HIV infection as part of combination prevention approaches?

**P:** People at substantial risk of HIV infection

**I:** Oral PrEP (containing tenofovir (TDF))

**C:** 1) placebo and 2) non-use of PrEP (or no use)

**O:** (1) HIV infection, (2) any adverse event, (3) any stage 3 or 4 adverse event, (4) drug resistance (among those initiating PrEP while acutely infected and among those who seroconvert after PrEP initiation), and 5) sexual and reproductive health outcomes, including 5a) hormonal contraception effectiveness, 5b) any adverse pregnancy event, 5c) condom use, and 5d) number of sexual partners

**Inclusion criteria**

To be included in the review, an article must meet the following criteria:

1. Randomized controlled trial or open-label extension/PrEP demonstration project evaluating the use of oral PrEP (containing tenofovir (TDF)) to prevent HIV infection among people at substantial risk of HIV infection.
2. Measured one or more of the key outcomes outlined in the PICO questions as listed above, comparing those randomized to oral PrEP versus placebo or comparing those receiving oral PrEP versus none use of PrEP (e.g., delayed use of PrEP).
3. Published in a peer-reviewed journal or presented as an abstract at a scientific conference between January 1, 1990 and January April 15, 2015.

No restrictions will be placed based on location of the intervention. No language restrictions will be used on the search. Articles in languages other than English will be translated where necessary for the main PICO questions, but not for the values and preferences review. Authors of studies will be contacted for clarification on study outcomes when needed.

Following the GRADE approach, if randomized controlled trials and high-quality observational studies present evidence for similar outcomes, results will be stratified by quality of evidence (e.g., randomized trials vs. observational studies) and presented in two separate rows of the GRADE table. Similarly, studies with different comparators (placebo or non-PrEP use) will be stratified in GRADE and included in two separate rows of the final GRADE table.

**Search strategy**

The following electronic databases will be searched using the date ranges January 1, 1990 to April 15, 2015: PubMed, CINAHL (Cumulative Index to Nursing and Allied Health Literature), and EMBASE. Iterative secondary reference searching will also be conducted on all studies included in the review until no new studies are identified. Further, selected experts in the field will be contacted to identify additional articles not identified through other search methods.

Abstracts from the following conferences will be searched from January 1, 1990 to April 15, 2015: International AIDS Conference (IAC), IAS Conference on HIV Pathogenesis, Treatment, and Prevention (IAS), and Conference on Retroviruses and Opportunistic Infections (CROI).

**Search terms**

The following terms will be entered into all computer databases:

(“pre-exposure prophylaxis” or “preexposure prophylaxis” or “antiretroviral prophylaxis” or “preexposure chemoprophylaxis” or chemoprevention or PrEP) AND (HIV OR AIDS)

These search terms will be used both for the main systematic review (PICO question) and for the values and preferences review.

**Screening abstracts**

Titles, abstracts, citation information, and descriptor terms of citations identified through the search strategy will be screened by a member of the senior study staff. Full text articles will be obtained of all selected abstracts and two independent reviewers will assess all full-text articles for eligibility to determine final study selection. Differences will be resolved through consensus.

Citations identified through computer database searching will be initially screened into the following categories:

- **Yes** – Used when the article appears to meet the inclusion criteria for the review.
- **Pull to check** – Used when the article may or may not meet the inclusion criteria, and the full text of the article must be reviewed before a final decision about inclusion can be made.
- **No** – Used when the article clearly does not meet the inclusion criteria for the review and no further consideration is necessary.
- **Values and Preferences** – Used when the article does not meet the inclusion criteria for the main review (PICO question), but does meet criteria for the values and preferences review (described below). Values and Preferences will be further categorized by the sub-population involved, including: 1) men who have sex with men; 2) transgender people; 3) women; 4) heterosexual men; 5) sex workers; 6) people who use drugs; 7) serodiscordant couples, 8) young people, etc.
- **Background** – Used when the article clearly does not meet the inclusion criteria for the review, but presents potentially relevant information. Background studies will be further subdivided into categories based on the type of information they provide, including: 1) review articles; 2) qualitative studies; 3) cost or cost-effectiveness analyses; 4) intervention descriptions without an evaluation component; and 5) drug/pharmacokinetic studies. An annotated bibliography of background articles will be created with citation information and abstracts.

**Data extraction and management**

Data will be extracted independently by two reviewers using standardized data extraction forms. Differences in data extraction will be resolved through consensus and referral to a senior study team member from WHO when necessary.

The following information will be gathered from each included study:

- Study identification: Author(s); type of citation; year of publication
- Study description: Study objectives; location; population characteristics; description of the intervention; study design; sample size; follow-up periods and loss to follow-up
- Outcomes: Analytic approach; outcome measures; comparison groups; effect sizes; confidence intervals; significance levels; conclusions; limitations

For randomized controlled trials, risk of bias will be assessed using the Cochrane Collaboration’s tool for assessing risk of bias (Cochrane Handbook, chapter 8.5 – Higgins & Green, 2011). This tool assesses random sequence generation (selection bias), allocation concealment (selection bias), blinding of participants and personnel (performance bias), blinding of outcome assessment (detection bias) blinding of outcome assessment (detection bias), incomplete outcome data addressed (attrition bias), incomplete outcome data, and selective reporting (reporting bias). Methodological components of studies will be assessed and classified as high, low, or uncertain risk of bias.

**Data Analysis**

Data will be analyzed according to coding categories and outcomes. Where there are multiple studies reporting the same outcome, meta-analysis will be conducted using random-effects models to combine odds ratios with the program Comprehensive Meta-Analysis (CMA). When meta-analysis is warranted, sensitivity analyses will be conducted in CMA to assess the robustness of findings by running the primary analysis with and without certain studies based on various characteristics, including overall adherence levels. Data will be summarized in GRADE tables, summary of finding tables, and risk/benefit tables.

Because this review covers multiple populations, drug regimens, drug dosing, and comparators, we have developed a list of *a priori* sub-group analyses to conduct. Prior to conducting these analyses, we will evaluate the credibility of each sub-group analysis based on the following characteristics as recommended by the GRADE process^3^:

- Is the subgroup variable a characteristic specified at baseline (in contrast with after randomization)?
- Is the subgroup difference suggested by comparisons within rather than between studies?
- Does statistical analysis suggest that chance is an unlikely explanation for the subgroup difference?
- Did the hypothesis precede rather than follow the analysis, and include a hypothesized direction that was subsequently confirmed?
- Was the subgroup hypothesis one of a small number tested?
- Is the subgroup difference consistent across studies and across important outcomes?
- Does external evidence (biological or sociological rationale) support the hypothesized subgroup difference?

The sub-group analyses to be evaluated include:

1. Sex of study participants (males vs. females)
2. Age of study participants (<25 vs. ≥25)
3. Primary mode of sexual HIV transmission (anal vs. vaginal/penile exposure)
4. Adherence to study drugs (based on overall drug detection levels in blood specimens)
5. PrEP dosing (daily vs. intermittent PrEP)
6. PrEP regimen (TDF vs. TDF-FTC)

**References**

1. WHO. Guidance on pre-exposure oral prophylaxis (prep) for serodiscordant couples, men and transgender women who have sex with men at high risk of HIV. Geneva: WHO; 2012.

2. WHO. Consolidated guidelines on HIV prevention, diagnosis, treatment and care for key populations. Geneva: WHO; 2014.

3. Guyatt, G.H., Oxman, A.D., Kunz, R., Woodcock, J., Brozek, J., Helfand, M. et al. GRADE guidelines: 7. Rating the quality of evidence—inconsistency. J Clin Epidemiol. 2011; 64: 1294–1302)

# Table 1S. Quality assessment of included studies (risk of bias)

|  | Risk of Bias Categories | | | | | |
| --- | --- | --- | --- | --- | --- | --- |
| Study | Random sequence generation (selection bias) | Allocation concealment (selection bias) | Blinding of participants and personnel (performance bias) | Blinding of outcome assessment (detection bias) | Incomplete outcome data addressed (attrition bias) | Selective reporting (reporting bias) |
| HPTN 067 (ADAPT) | Low | Low | Low | Low | Low | Low |
| Bangkok Tenofovir Study | Low | Low | Low | Low | Low | Uncertain |
| CDC Safety Study | Low | Low | Low | Low | Low | Uncertain |
| FEM-PrEP^a^ | Low | Low | Low | Low | Low | Low |
| Ipergay | Low | Low | Low | Low | Low | Uncertain |
| iPrEx | Low | Low | Low | Low | Low | Low |
| IAVI Kenya Study | Low | Low | Low | Low | Low | Low |
| IAVI Uganda Study | Low | Low | Low | Low | Low | Low |
| Partners PrEP | Low | Low | Low | Low | Low | Low |
| Partners PrEP Study Continuation | Low | Low | Low | Low | Low | Low |
| Project PrEPare | Low | Low | Uncertain | Uncertain | Low | Uncertain |
| PROUD | Low | Low | Uncertain | Uncertain | Low | Low |
| TDF2 | Low | Low | Low | Low | Low | Low |
| VOICE^a^ | Low | Low | Low | Low | Low | Uncertain |
| West Africa Study | Low | Low | Low | Low | Low | Low |

^a^Studies reported less than 40% adherence to the study drug; therefore, there is risk that these studies are not able to fully answer the research questions the studies were designed to answer, including their ability to assess the efficacy of PrEP. However, because no trial demonstrated perfect adherence, this risk is implicit in all trials, but heightened in trials with lowest adherence.

# Figure 1S. Forest plot for overall analysis of PrEP and HIV Infection

Favors Placebo

Favors PrEP

# Table 2S. Drug resistant mutations identified in each study among seroconverters

| **Study** | | **Acute HIV Infections at Enrollment** | | | **Post-Randomization HIV Infections** | | |
| --- | --- | --- | --- | --- | --- | --- | --- |
|  | Study Arm | No. of sero-converters | TDF Mutations | FTC Mutations | No. of sero-converters | TDF Mutations | FTC Mutations |
| **Bangkok Tenofovir Study^a^** | PrEP: TDF | 0 | 0 | NR | 17 | 0 | NR |
|  | Placebo | 2 | 0 | NR | 33 | 0 | NR |
| **FEM-PrEP** | PrEP: TDF-FTC | 1^b^ | 0 | 0 | 33^c^ | 0 | **4**^d^ |
|  | Placebo | 1^b^ | 0 | 0 | 35 | 0 | 1 |
| **iPrEx** | PrEP: TDF-FTC | 2 | 0 | 2 | 48 | 0 | 0 |
|  | Placebo | 8 | 0 | **1** | 83 | 0 | 0 |
| **Partners PrEP** | PrEP: TDF | 5 | 1 | 0 | 15 | 0 | 0 |
|  | PrEP: TDF-FTC | 3 | 0 | 1 | 12 | 0 | 0 |
|  | Placebo | 6 | 0 | 0 | 51 | 0 | 0 |
| **TDF2** | PrEP: TDF-FTC | 1 | **1**^e^ | **1**^e^ | 9 | 0 | 0 |
|  | Placebo | 2 | 0 | 0 | 24 | 0 | 0 |
| **VOICE** | PrEP: TDF | 5 | 0 | 0 | 58 | 0 | 0 |
|  | PrEP: TDF-FTC | 9 | 0 | 2 | 55 | 0 | 1 |
|  | Placebo | 1 | 0 | 0 | 60 | 0 | 0 |
| **TOTAL (overall)** | | **46** | **2** | **6** | **533** | **0** | **6** |
| **TOTAL (by study arm)** | FTC/TDF | 16 | **1** | **5** | **157** | **0** | **5** |
|  | TDF | 10 | **1** | **0** | **90** | **0** | **0** |
|  | Placebo | 20 | **0** | **1** | **286** | **0** | **1** |

**^a^** RNA was amplified in specimens from 49 of 52 HIV-positive participants for molecular genotyping

^b^ Data abstracted from 2012 presentation by Liegler et al. at drug resistance workshop

**^c^** Three HIV infections identified in the TDF-FTC group occurred within 12 weeks of enrollment. Investigators note the possibility that these individuals could have been acutely infected during enrollment.

**^d^** One participant with FTC-mutation had not accessed FTC/TDF for 48-weeks prior to seroconversion due to early toxicity.

^e^ Participant assigned to TDF-FTC arm was falsely screened negative for HIV infection at enrollment and was diagnosed with HIV infection at the month 7 study visit. Mutations for both TDF and FTC were detected.

# Table 3S. Meta-analysis results for PrEP and drug resistance among seroconverters

| Type of Mutation | Timing of HIV Infection | PrEP regimen | No. of studies | Pooled RR | 95% CI | p-value |
| --- | --- | --- | --- | --- | --- | --- |
| Any mutation  (TDF or FTC) | Acute infection at enrolment | TDF and FTC/TDF | 4^a^ | 3.34 | (1.11-10.06) | 0.03 |
| Any mutation  (TDF or FTC) | Post-randomization | TDF and FTC/TDF | 2^b^ | 3.14 | (0.53-18.52) | 0.21 |
| FTC (M184I; M184V) | Acute infection at enrolment | TDF and FTC/TDF | 4^a^ | 3.12 | (1.03-9.46) | 0.05 |
| FTC (M184I; M184V) | Post-randomization | TDF and FTC/TDF | 2^b^ | 3.91 | (0.66-23.07) | 0.13 |
| FTC (M184I; M184V) | Acute infection at enrolment | FTC/TDF | 4^a^ | 3.72 | (1.23-11.23) | 0.02 |
| FTC (M184I and M184V) | Post-randomization | FTC/TDF | 2^b^ | 3.91 | (0.66-23.07) | 0.13 |
| TDF (KR65; K70E) | Acute infection at enrolment | TDF and FTC/TDF | 2^d^ | 3.39 | (0.46-25.05) | 0.23 |
| TDF (KR65; K70E) | Post-randomization | TDF and FTC/TDF | 0 | -- | -- | -- |
| TDF (KR65; K70E) | Acute infection at enrolment | TDF | 1^e^ | 3.50 | (0.17-70.94) | 0.42 |
| TDF (KR65; K70E) | Post-randomization | TDF | 0 | -- | -- | -- |

^a^ iPrEx, Partners PrEP, TDF2, and VOICE

^b^ FEM-PrEP and VOICE

^d^ Partners PrEP and TDF2

^e^Partners PrEP

# Table 4S. Summary of pregnancies rates in studies reporting contraceptive effectiveness

| Study | Contraceptive Method | PrEP  Pregnancies | PrEP person-years | Placebo Pregnancies | Placebo person-years | Rate ratio^a^ | 95% confidence interval^a^ |
| --- | --- | --- | --- | --- | --- | --- | --- |
| FEM-PrEP | Any method | 69 | 602.9 | 48 | 620.7 | 1.48 | (1.02-2.14) |
|  | COCs | 60 | 171.1 | 43 | 153.9 | 1.26 | (0.85-1.86) |
|  | Injectables | 9 | 402.3 | 5 | 448.2 | 2.01 | (0.67-5.98) |
| Partners PrEP | Any method | 67 | 924.2 | 28 | 508.1 | 1.32 | (0.85-2.05) |
|  | COCs | 37 | 209.3 | 11 | 108.7 | 1.75 | (0.89-3.42) |
|  | Implants | 1 | 150.6 | 0 | 79.7 | 1.59 | (0.07-39.0) |
|  | Injectables | 29 | 564.3 | 17 | 319.7 | 0.97 | (0.53-1.76) |

^a^Rate ratios and confidence intervals calculated using CMA v3.0

# Table 5S. Summary of results for PrEP and hormonal contraception effectiveness

| Study | Comparison | Adjusted HR | p-value |
| --- | --- | --- | --- |
| FEM-PrEP^a^ | Risk of pregnancy comparing PrEP to placebo *(adjusted for contraceptive method, site, and age)* | 1.2 (0.9 to 1.8) | 0.20 |
| Partners PrEP –  Combined Oral Contraceptives (COCs)^b^ | Risk of pregnancy comparing COCs to no contraception among PrEP group  Risk of pregnancy comparing COCs to no contraception among placebo group  **P-value for difference in HRs by arm** | 0.96 (0.58-1.58)  0.55 (0.26-1.19) | 0.87  0.13  **0.24** |
| Partners PrEP –  Injectables^b^ | Risk of pregnancy comparing injectables to no contraception among PrEP group  Risk of pregnancy comparing injectables to no contraception among placebo group  **P-value for difference in HRs by arm** | 0.26 (0.16-0.41)  0.19 (0.10-0.37) | <0.0001  <0.0001  **0.47** |

^a^ Analysis from: Callahan R, Nanda K, Kapiga S, et al. Pregnancy and contraceptive use among women participating in the FEM-PrEP trial.

*Journal of Acquired Immune Deficiency Syndromes* 2015; **68**(2): 196-203.

^b^ Analysis from: Murnane PM, Heffron R, Ronald A, et al. Pre-exposure prophylaxis for HIV-1 prevention does not diminish the pregnancy prevention effectiveness of hormonal contraception. *AIDS* 2014; **28**(12): 1825-30.

# Table 6S: Meta-analysis results for effect of PrEP on pregnancy-related adverse events

| Analysis | No. of studies | Risk Ratio (95% CI) | p-value | I^2^ |
| --- | --- | --- | --- | --- |
| RCTs comparing PrEP to placebo | | | | |
| Overall^a,b^ | 2 | 1.25 (0.64-2.45) | 0.52 | 64.5 |
| PrEP use  Low  High | 1  1 | 1.96 (0.90-4.3)  0.96 (0.72-1.28) | 0.09  0.80 | n/a  n/a |
| Drug Regimen  TDF  TDF-FTC | 1  2 | 0.77 (0.55-1.10)  1.35 (0.93-1.95) | 0.15  0.11 | n/a  16.7 |
| Observational studies | | | | |
| Partners PrEP OLE | After unmasking, frequency of pregnancy loss was 37.5% for FTC/TDF and 36.7%for TDF alone (difference, 0.8%; 95%CI, −16.8%to 18.5%; P = .92). | | | |

^a^ For FEM-PrEP authors note the higher pregnancy-related adverse event rate in the FTC/TDF group (P = 0.04), but also note there were more pregnancies in this group as compared with the placebo group (IR=11.2 per 100 person-years vs. 7.5 per 100 person-years, respectively).

^b^ Both trials tested participants for pregnancy at monthly study visits. Therefore, participants were mostly only receiving PrEP during the initial weeks of pregnancy.

# Table 7S: Summary of changes on condom use across PrEP studies

| Study | Behavioral Measure | Outcome |
| --- | --- | --- |
| RCTs: Comparisons between PrEP and placebo groups | | |
| Fem-PrEP | Sex acts without a condom used | Modest but significant reduction (mean reduction= 0.46; P<0.001) comparing last follow-up visit to 7 days prior to enrollment. |
| iPrEx | Percent of receptive anal partners with which condoms used | Baseline:50.38% in TDF-FTC; 51.04% in placebo  Follow-up (week 132) : 73.98% in TDF-FTC: 83.64% in placebo  Wald test treatment by visit interaction: p=0.36. |
| Partners PrEP | Having sex without a condom with HIV-positive partners in prior month | Baseline: 27%  Follow-up (12 months): 13%  Follow-up (24 months): 9%  (similar across study groups) |
| TDF2 | Protected sex episodes with main/ most recent casual partner | Similar at enrollment (p=0.66) and remained stable over time  TDF-FTC: 81.4% [range, 76.6 to 86.4]  Placebo: 79.2% [range, 71.6 to 87.6] |
| West Africa PrEP Study | Condom use at last sex | Screening: 52%  12-month follow-up: 95% (for acts in past 7 days) |
| RCTs: Comparisons between PrEP and no PrEP | | |
| CDC Safety Study | Unprotected Anal Sex (UAS)  Unprotected anal sex with HIV positive/ unknown status partner (UASPU) | •Decrease from baseline: (57%) to months 3–9 (48%, P = 0.001) and months 12–24 (52%, P = 0.03). Change in proportion similar between immediate vs. delayed arms (P=0.15)  •No statistical difference in UAS episodes between immediate vs. delayed arms during months 3–9 (P = 0.10) and no significant change when delayed group initiated study drug (P = 0.42)  •Mean UASPU remained stable or decreased during follow-up [2.02 at baseline vs. 1.51 during months 3–9(P = 0.22) and 1.37 during months 12–24 (P = 0.05)] |
| Project PrEPare | Male-to-male unprotected anal sex acts | •No significant differences among the 3 treatment groups across visits.  •Insignificant trend from baseline to week 24 of decreasing unprotected anal sex acts across all treatment arms. |
| PROUD | Incident STIs (proxy for unprotected sex) | Proportion with confirmed rectal chlamydia/gonorrhea) was similar in immediate (29%) and delayed (27%) (P=0.50) arms. |
| Observational studies and RCTs making longitudinal comparisons between pre/post PrEP use | | |
| iPrEx OLE | Non-condom receptive anal intercourse  Syphilis incidence (proxy for unprotected sex) | •Decreased from 34% (377/1115) to 25% (232/926) among PrEP recipients (p=0.006), and from 27% (101/369) to 20% (61/304) among non-recipients (p=0.03).  •Decreases in non-condom receptive anal intercourse, non-condom insertive anal intercourse, were similar across groups (p=0.95 and p=0.56)  •Syphilis incidence was similar among PrEP recipients and non-recipients (7.2 infections per 100 patient-years vs 5.4 infections per 100 patient-years, HR 1.35, 95 CI 0.83–2.19). |
| Partners PrEP Study Continuation^a^ | Unprotected sex | •Trend toward decreasing frequency of unprotected sex with study partner during study before unmasking, and after unmasking no significant changes in the immediate level (p=0∙66) or trend (p=0∙25) of unprotected sex  •Significant increase in frequency of unprotected sex over time with outside partners (p=0∙04). Consequence of this change in trend was a small difference in the estimated vs counterfactual annual average total frequency of unprotected sex acts |

^a^ Study contains data from placebo-controlled RCT and Continuation Study comparing TDF to FTC/TDF (no placebo).

# Table 8S: Summary of changes in number of sexual partners across PrEP studies

| Study | Behavioral Measure | Outcome |
| --- | --- | --- |
| RCTs: Comparisons between PrEP and placebo groups | | |
| Fem-PrEP | Sex acts without a condom used | Modest but significant reduction (mean reduction= 0.46; P<0.001) comparing last follow-up visit to 7 days prior to enrollment. |
| iPrEx | Percent of receptive anal partners with which condoms used | Baseline:50.38% in TDF-FTC; 51.04% in placebo  Follow-up (week 132) : 73.98% in TDF-FTC: 83.64% in placebo  Wald test treatment by visit interaction: p=0.36. |
| Partners PrEP | Having sex without a condom with HIV-positive partners in prior month | Baseline: 27%  Follow-up (12 months): 13%  Follow-up (24 months): 9%  (similar across study groups) |
| TDF2 | Protected sex episodes with main/ most recent casual partner | Similar at enrollment (p=0.66) and remained stable over time  TDF-FTC: 81.4% [range, 76.6 to 86.4]  Placebo: 79.2% [range, 71.6 to 87.6] |
| West Africa PrEP Study | Condom use at last sex | Screening: 52%  12-month follow-up: 95% (for acts in past 7 days) |
| RCTs: Comparisons between PrEP and no PrEP | | |
| CDC Safety Study | Unprotected Anal Sex (UAS)  Unprotected anal sex with HIV positive/ unknown status partner (UASPU) | •Decrease from baseline: (57%) to months 3–9 (48%, P = 0.001) and months 12–24 (52%, P = 0.03). Change in proportion similar between immediate vs. delayed arms (P=0.15)  •No statistical difference in UAS episodes between immediate vs. delayed arms during months 3–9 (P = 0.10) and no significant change when delayed group initiated study drug (P = 0.42)  •Mean UASPU remained stable or decreased during follow-up [2.02 at baseline vs. 1.51 during months 3–9(P = 0.22) and 1.37 during months 12–24 (P = 0.05)] |
| Project PrEPare | Male-to-male unprotected anal sex acts | •No significant differences among the 3 treatment groups across visits.  •Insignificant trend from baseline to week 24 of decreasing unprotected anal sex acts across all treatment arms. |
| PROUD | Incident STIs (proxy for unprotected sex) | Proportion with confirmed rectal chlamydia/gonorrhea) was similar in immediate (29%) and delayed (27%) (P=0.50) arms. |
| Observational studies and RCTs making longitudinal comparisons between pre/post PrEP use | | |
| iPrEx OLE | Non-condom receptive anal intercourse  Syphilis incidence (proxy for unprotected sex) | •Decreased from 34% (377/1115) to 25% (232/926) among PrEP recipients (p=0.006), and from 27% (101/369) to 20% (61/304) among non-recipients (p=0.03).  •Decreases in non-condom receptive anal intercourse, non-condom insertive anal intercourse, were similar across groups (p=0.95 and p=0.56)  •Syphilis incidence was similar among PrEP recipients and non-recipients (7.2 infections per 100 patient-years vs 5.4 infections per 100 patient-years, HR 1.35, 95 CI 0.83–2.19). |
| Partners PrEP Study Continuation^a^ | Unprotected sex | •Trend toward decreasing frequency of unprotected sex with study partner during study before unmasking, and after unmasking no significant changes in the immediate level (p=0.66) or trend (p=0.25) of unprotected sex  •Significant increase in frequency of unprotected sex over time with outside partners (p=0.04). Consequence of this change in trend was a small difference in the estimated vs counterfactual annual average total frequency of unprotected sex acts |

^a^ Study contains data from placebo-controlled RCT and Continuation Study comparing TDF to FTC/TDF (no placebo).

# Table 9S. Drug detection among seroconverters and HIV-negative controls randomized to active study drug

|  | **Seroconverters assigned to active study drug** | | **HIV-negative controls assigned to active study drug** | |
| --- | --- | --- | --- | --- |
| **Study** | Proportion with detectable levels of TDF or FTC | Percent with detectable levels of TDF or FTC | Proportion with detectable levels of TDF or FTC | Percent with detectable levels of TDF or FTC |
| **Bangkok Tenofovir Study** | 5/39 | 39% | 93/138 | 67% |
| **CDC Safety Study** | Not reported | Not reported | 44/47^a^ | 94% |
| **FEM-PrEP^b^** | 7/27 (beginning of infection window)  7/33 (end of window)  4/27 (both visits) | 26%  21%  15% | 27/48 (beginning of infection window)  35/95 (end of window)  19/78 (both visits) | 35%  37%  15% |
| **iPrEx** | 3/34 | 1% | 22/43 | 51% |
| **Partners PrEP Study** | 29 | 31% | 902 | 82% |
| **TDF2** | 2/4 | 50% | 55/69 | 80% had TDF  81% had FTC |
| **VOICE^c^** | 12/51 (TDF group)  17/57 (FTC-TDF group) | 24%  30% | 50/145 (TDF group)  45/132 (TDF-FTC group) | 34%  22% |

^a^ This measure includes drug detection using plasma. The study also assessed Tenofovir (TFV) detection using peripheral blood mononuclear cells (PBMC) and hair samples. The three specimens that did not have detectable TFV levels in plasma did have detectable TFV levels in either hair and/or PBMC samples. Results from plasma are presented here as this method was most similar to methods used by other studies.

^b^ FEM-PrEP defined drug detection as ≥10 nanograms per milliliter.

^c^ Results from the VOICE trial presented here come from the Marrazzo et al., 2015 Supplementary Appendix Table S6B and reflect the number of women in each study arm that had detectable drug levels at their first quarterly visit. Marrazzo et al. also reports that among a random sample of participants assigned to active study drug (Ns not reported), Tenofovir was detected in 30% of participants assigned to TDF and in 29% of participants assigned to TDF-FTC. For meta-analysis, the reported proportion of participants from the random sample was used as the marker of overall trial-level adherence.
